# Exercise for myeloma patients with bone disease: a scoping review

**DOI:** 10.1007/s00520-026-10958-7

**Published:** 2026-07-13

**Authors:** Joanne Land, Orla McCourt, Charalampia Kyriakou, Abigail Fisher, Kwee Yong

**Affiliations:** 1 Department of Haematology, University College London Hospitals National Health Service Foundation Trust, London, United Kingdom; 2 UCL Department of Behavioural Science and Health, 1-19 Torrington Place, London, WC1E 7HB United Kingdom; 3 Department of Haematology, University College London (UCL) Cancer Institute, London, United Kingdom

**Keywords:** Multiple myeloma, Exercise, Bone

## Abstract

**Purpose:**

Exercise is considered safe for patients with myeloma, but evidence specific to multiple myeloma bone disease (MMBD) is unclear. Scoping review aims: 1) Review the literature on exercise interventions for myeloma patients that included those with MMBD 2) Map reporting of safety, assessment and intervention characteristics 3) Identify bone-related outcomes measuring changes in MMBD 4) Highlight research priorities.

**Method:**

Following the Joanna Briggs Institute framework, a comprehensive search of PubMed, OVID-MEDLINE, and CINAHL between 1946 to October 2025 was conducted.

**Results:**

Twelve studies met inclusion criteria, though none focused exclusively on MMBD patients. Reporting of MMBD characteristics varied; across 346 participants, prevalence ranged from 69–86%. There were 7 exercise-related adverse events, but it was not reported if they occurred in MMBD patients. Most studies used medical clearance, imaging, or standardized tools to assess participants’ suitability for exercise. However, procedures varied between studies and haematological considerations were generally not utilised. Patients were commonly excluded if they had spinal instability, spinal cord compression or were at risk of a fracture. Interventions occurred across all disease stages and commonly involved tailored, supervised/ unsupervised aerobic and resistance exercise, led by physiotherapists. However, practitioner experience, exercise modifications for MMBD and bone-related outcomes were infrequently reported, limiting clinical application.

**Conclusion:**

Exercise appears safe for MMBD patients under certain exclusion criteria, but inconsistent reporting of adverse events in MMBD patients, MMBD characteristics, assessments, and exercise adaptations limits clinical translation, highlighting the need for targeted trials with only MMBD patients and bone-specific outcomes.

## Introduction

Multiple myeloma is an incurable blood cancer marked by plasma cell proliferation in the bone marrow. It can cause anaemia, hypercalcaemia, renal impairment and immunoparesis [1]. However, bone disease is one of its most common and debilitating features, affecting up to 80% of newly diagnosed patients [2].

Multiple myeloma bone disease (MMBD) results from dysregulated bone remodelling, where osteoclast activity is increased and osteoblast activity suppressed [3]. Leading to accelerated bone resorption, skeletal fragility and a high risk of skeletal-related events (SREs), including fractures, spinal cord compression and the need for surgery or radiotherapy [1].

Fractures occur in more than half of patients, and spinal cord compression affects up to 30% [2, 4]. These complications contribute to poorer survival outcomes [5], reduced quality of life [6], impaired physical and social functioning [6, 7] and higher healthcare costs [8].

Exercise has demonstrated benefits for myeloma patients including improvements in clinical levels of fatigue [9], exercise capacity [9], mood [10], strength [9, 11] and higher levels of physical activity associated with improved quality of life [12].

However, evidence specific to MMBD patients is limited. Although recent systematic reviews have reported exercise to be both safe and feasible for individuals with myeloma [13]. Concerns about SREs in the presence of MMBD often results in clinicians avoiding exercise recommendations [14], despite evidence from osteoporosis showing that mechanical loading during exercise can stimulate bone formation [15]. While consensus-based guidelines exist for other cancers, there are currently no dedicated exercise guidelines for MMBD, contributing to uncertainty in clinical practice. It highlights the need for greater support and guidance to help healthcare professionals determine what assessment procedures and exercises are safe for this higher risk group, and to clarify the efficacy of exercise in improving bone health [14].

Existing reviews have examined the safety and general outcomes of exercise in myeloma patients [13, 16], however, none have specifically addressed those with MMBD and exercise guidance is absent from existing MMBD practice recommendation guidelines [3, 17]. Scoping reviews can systematically map literature and identify gaps in knowledge [18]. Therefore, this review aims to map the current literature on exercise interventions that include patients with MMBD, evaluating clinical considerations, safety, outcomes and exercise prescription approaches to support future research and improve guidance for this higher-risk group.

## Objectives

1) Conduct a systematic search of the literature on exercise management for MMBD patients 2) Describe the assessment procedures, characteristics of the exercise interventions used and reporting of safety, 3) Identify bone-related outcomes measuring changes in MMBD, 4) Identify research gaps.

## Methods

### Approach

A structured scoping review was conducted using the framework developed by the Joanna Briggs Institute [19] and the PRISMA Extension for Scoping review (PRISMA-ScR) was used to guide reporting [18]. This was to identify, describe and map the peer-reviewed literature surrounding exercise for MMBD. A protocol for this scoping review was not published.

### Information sources

To identify relevant published peer reviewed literature the following bibliographic databases were searched from 1946 – October 2025: Pubmed, OVID-MEDLINE and CINAHL Plus. The author JL developed and executed the search strategy and details can be found in appendix 1.

### Search terms

The electronic search used a combination of MeSH and free text terms for “multiple myeloma*”, “exercise”, “physical fitness”, “physical activity” and “rehabilitation”.

### Selection of sources of evidence

Initially retrieved records were downloaded into a single reference list using EndNote (version 21) where duplicates were removed. Titles and abstracts were screened by JL according to pre-defined inclusion and exclusion criteria. The reference lists of retrieved studies were searched to identify further relevant publications. The full text of included studies was sourced and read in full according to the inclusion/exclusion criteria.

In order to scope the majority of literature while ensuring relevance to the research question, exclusion criteria were kept minimal and included: 1) non-human subjects, 2) grey literature sources, and 3) non-primary research (e.g., reviews, conference abstracts, editorial letters).

### Included studies

- The primary population are multiple myeloma patients with bone disease/lesions at any stage of treatment, if it is a mixed sample of cancer patients ≥ 50% have myeloma.
- Primary intervention was any exercise intervention comprising ≥ 1 session of structured exercise either supervised or unsupervised.
- Randomised controlled trials (RCTs) in which interventions consisted of exercise versus control or usual treatment or comparator interventions, observational and single arm studies were included.
- Outcomes reported – Adverse events (AE) related to exercise, outcomes indicating change in MMBD.

### Data extraction

A data extraction form was developed by JL with prespecified categories. The following characteristics were included: (i) Study identifiers (author, publication year) (ii) Study characteristics (country, study design, data collection period), study population (number of participants, stage disease, sex, age, extent MMBD involvement, bone modifying agent, time from diagnosis, history of SRE’s) (iii) Assessment (procedures to commence exercise, exclusion criteria) (iv) Characteristics of the intervention (setting, frequency, duration, type of exercise, intensity, health care professional delivering intervention and their experience, MMBD exercise adaptations including frequency, reason and how adapted) (v) Safety (number of non-exercise and exercise related AEs including consequences) and (vi) Outcomes (results indicating change in MMBD).

### Data analysis

All of the eligible studies were reviewed and details of relevant characteristics were entered. Table 1 reports on the study identifiers, study characteristics, population and safety. Table 2 presents the characteristics of the intervention and Table 3 reflects the assessment procedures and exercise adaptions utilised. Table 4 reports on outcomes indicating change in MMBD.

**Table 1:** Study identifiers, study characteristics, study population and safety

Reference	Country	Study design	Data collection	Study population	Clinical Characteristics	Adverse Events
Groenveldt et al. (2013)	UK	Single arm pre & post test	Oct 2006—Dec 2007	49 participants Post completion MM treatment **Time diagnosis:** NR **Male:** *n* = 26 (58%) **Age:** Median 61yrs (range 46–74)	**Significant MMBD**: *n* = 23 (51%) prev VCF or fractures long bones **Bone modifying agent:** NR **SREs:** Surgery: 15% SCC: NR RT: NR Fractures: 47%	**SAE exercise related:** 0 **SAE non-exercise related:** NR **Non-SAE exercise related:** 0 **Non-SAE non-exercise related:** NR
Shallwani et al. (2015) [29]	Canada	Retrospective real world data	Jan 2011—Dec 2013	41 participants (compliant: 29 non compliant: 12) During MM Treatment **Time diagnosis**: NR **Male:** compliant 75.9%: Non compliant 66.7% **Age:** Mean: compliant 61.5yrs: Non compliant 59.9yrs	**MMBD:** *n* = 33 bone lesions: compliant 75.9% non compliant 91.7% Bone lesion pain: compliant 88.6% non compliant 75% **Bone modifying agent:** NR **SREs:** Surgery: 40.8% SCC: 16.7 RT: 53.4% Fractures: *n* = 23	**SAE exercise related:** 0 **SAE non-exercise related:** NR **Non-SAE exercise related:** 0 **Non-SAE non-exercise related:** NR
Larsen et al. (2019) [21]	Denmark	RCT	June 2015—June2016	30 participants (17 IG: 13 CG) Newly diagnosed MM (1 week) **Time diagnosis**: NR **Male:** IG *n* = 14 (82%): CG *n* = 9 (69%) **Age:** mean (± SD): IG 69yrs (9.7): CG 67yrs (15.3)	**MMBD:** IG *n* = 14 (82%): CG *n* = 6 (46%) Restrictions testing/exercise: IG 9 (64%) CG 1 (17%) **Bone modifying agent:** NR **SREs:** Surgery: NR SCC: NR RT: NR Fractures: NR	**SAE exercise related:** 0 **SAE non-exercise related:** NR **Non-SAE exercise related:** 0 **Non-SAE non-exercise related:** 1 × Pain: 1 × dizziness **Outcome**: Stop 1 × exercise session (*n* = 2)
Koutoukidis & Land et al. (2020) [9]	UK	Zelen/RCT	June 2014—Nov 2016	131 participants (89 IG: 42 CG) Stable MM disease for 6 wks **Time diagnosis:** NR **Male:** IG *n* = 48 (54%): CG *n* = 24 (57%) **Age:** Median (range): IG 64yrs (35–86): CG 63yrs (40–80)	**MMBD:** IG *n* = 61 (69%): CG *n* = 29 (69%) **Bone modifying agent:** NR **SREs:** Surgery: 18% SCC: NR RT: 26% Fractures: NR	**SAE exercise related: 0** **SAE non-exercise related:** 0 **Non-SAE exercise related:** 1 × Hip pain **Outcome:** spontaneously improved (*n* = 1) **Non-SAE non-exercise related:** 4 × LBP? cause **Outcome:** NR (*n* = 4)
Wefelnberg et al. (2022) [30]	Germany	Retrospective real world data	Jan 2012—Nov 2019	26 participants Before & during treatment **Time diagnosis**: NR **Male:** *n* = 16 (61.5%) **Age:** Mean (± SD) 64yrs (± 8)	**MMBD** focal *n* = 3 (11.5%): Disseminated *n* = 18 (69.2%): Fractures *n* = 11 (42.3%) **Bone modifying agent:** NR **SREs:** Surgery: 42.3% SCC: NR RT: 50% Fractures: 42.3%	**SAE exercise related:** 0 **SAE non-exercise related**: 0 **Non-SAE exercise related:** 0 **Non-SAE non-exercise related:** 0
Purdy et al. (2022) [27]	Canada	Single arm pre & post test	Sept 2020—March 2021	29 participants (i) Transplant ineligible, first-line treatment (ii) transplant eligible, > 3 months post-transplantation (iii) relapsed/recurrent myeloma with ≥ 1 prior line of treatment **Time diagnosis:** Median 35mths (range 9–164) **Male:** *n* = 14 (50%) **Age:** Mean (± SD) 65yrs (8.4)	**MMBD:** *n* = 24 (86%) Location Thoracic (57%): Lumbar (32%): Pelvis (32%): Femur (32%): Skull (25%): Humerus (14%): Cervical spine (7%): other (11%) Extent of bone disease**:** Minor (1 location) 21%: Moderate (2 locations) 32%: Major (≥ 3 locations) 32% **Bone modifying agent:** NR **SREs:** Surgery: 29% SCC: NR RT: 46% Fractures: NR	**SAE exercise related:** 0 **SAE non-exercise related:** 0 **Non-SAE exercise related:** 2 × Mild back pain (seated bridging/low impact jumping jack): 1 × moderate back pain (bridging): 1 × Mod/Sev back pain (hip flexor stretch) **Outcome:** Mild pain ≤ few days (*n* = 2): Mod pain 6 d pause (*n* = 1): Mod/Sev pain 7 d pause (*n* = 1) **Non-SAE non-exercise related**: 1 × spinal fracture fall: 1 × mod back pain slip on ice: 1 × arrhythmia **Outcome:** spinal fracture aborted programme (*n* = 1): mod back pain 4 d pause (*n* = 1): arrythmia 10 d pause (*n* = 1)
McCourt et al. (2023) [23]	UK	RCT	June 2019—Oct 2020	50 participants (23 IG: 27 CG) MM transplant eligible **Time diagnosis:** Median (IQR) IG 7mths (6,11): CG 6mths (5,10) **Male:** IG *n* = 13 (57%): CG *n* = 18 (67%) **Age:** mean (± SD): IG 59.3yrs (9.4): CG 61.3yrs (8.7)	**MMBD:** IG *n* = 18 (78%): CG *n* = 20 (74%) Location MMBD: Axial IG *n* = 11 (48%): CG *n* = 15 (56%): Axial & peripheral IG *n* = 7 (30%): CG *n* = 5 (19%) Symptoms bone disease: MM pain IG *n* = 12 (52%): CG *n* = 13 (48%): Restricted mobility: IG *n* = 3 (13%): CG n = 1 (4%) non MM pain: CG *n* = 2 (9): CG *n* = 4 (15%) **Bone modifying agent:** NR **SREs:** Surgery: 13% SCC: NR RT: 17% Fractures: NR	**SAE exercise related:** 0 **SAE non-exercise related:** 0 **Outcome:** NR **Non-SAE exercise related:** (IG)? 1 × dizziness: (CG) 1 × Spinal** fracture** **Outcome:** NR **Non-SAE non-exercise related:** 0
Nicol et al. (2023)	Australia	RCT	April 2019—Sept 2020	60 participants (29 IG: 30 CG) Any stage MM **Time diagnosis:** NR **Male:** IG *n* = 21 (72.4%): CG *n* = 25 (83.3%) **Age:** mean (± SD): IG 67.1yrs (9.1): CG 62.9yrs (8.6)	**MMBD:** bone lesions IG *n* = 25 (86.2%): CG *n* = 23 (76.7%) Location: Spine: IG *n* = 21 (72.4%) CG *n* = 21 (70%) Pelvis: IG *n* = 11 (37.9%) CG *n* = 7 (23.3%) Ribs: IG *n* = 10 (34.5%) CG *n* = 6 (20%) Femur: IG *n* = 3 (10.3%) CG *n* = 7 (23.3%) Humerus: IG *n* = 3 (10.3%) CG *n* = 3 (10%) Other: IG *n* = 6 (20.7%) CG *n* = 7 (23.3%) Skeletal complications: Osteoporosis (T score ≤ − 2.5 at femoral neck): IG n = 2 (6.9%) CG n = 2 (6.7%) Osteopenia (T score < − 2.5 to < − 1.0 at femoral neck): IG n = 17 (58.6%) CG n = 16 (53.3%) **Bone modifying agent:** IG 24 (82.8%): CG 22 (73.3% **SREs:** Surgery: NR SCC: NR RT: NR Fractures: reported with lesions	**SAE exercise related:** 0 **SAE non-exercise related:** 0 **Non-SAE exercise related:** 0 **Non-SAE non-exercise related:** 1 × hypotensive episode **Outcome:** Continued with programme (*n* = 1)
Dapunt et al. (2023) [28]	Germany	Retrospective real world data	July 2021—Jan 2023	100 participants Any stage MM **Time diagnosis:** Median 25.5mths (range 2–159) **Male:** *n* = 62 (62%) **Age:** Median (range) 61.5yrs (27–83)	**MMBD:** *n* = 75 (75%) with ≥ 7 osteolytic lesions Location: SINS potentially unstable or unstable spine: (60%) Fracture risk (MIRELs score 8): Femur: 7patients Humerus: 1 patient, Fracture risk (Mirels score ≥ 9): Femur & Humerus 2 patients **Bone modifying agent:** NR **SREs:** Surgery: NR SCC: NR RT: NR Fractures: NR	**SAE exercise related:** 0 **SAE non-exercise related:** NR **Non-SAE exercise related:** 0 **Non-SAE non-exercise related:** NR
Larsen et al. (2024) [22]	Denmark	RCT	June 2015—July 2019	86 participants (44 IG: 42 CG) Newly diagnosed MM (1 week) **Time diagnosis:** NR **Male:** IG *n* = 26 (59%): CG *n* = 23 (55%) **Age:** Mean (± SD): IG 68.2yrs (9.1): CG 66.3yrs (11.5)	**MMBD:** IG *n* = 35 (80%): CG *n* = 29 (69%) **Denosumab:** IG 39 (89%) CG 41 (98%) **SREs:** Surgery: NR RT: 0% SCC: NR Fractures: 2% vertebral 0% non vertebral	**SAE exercise related: 0** **SAE non-exercise related:** 20 × hospital **Non-SAE exercise related:** 1 × excessive load **Outcome:** see below, doesn’t specify outcome **Non-SAE non-exercise related:** 4 × NR **Outcome**: aborted programme (*n* = 3): medical review (*n* = 1): other (*n* = 1)
Kollikowski et al. (2025) [20]	Germany	RCT	May 2022—March 2023	20 participants (12 IG: 8 CG) Any stage MM **Time diagnosis:** NR **Male:** IG *n* = 6 (50%): CG *n* = 4 (50%) **Age:** Mean: IG 59.3yrs: CG 63.1yrs	**MMBD** pts completed training: IG *n* = 7 out of 9 (77%): CG *n* = 7 out of 7 (100%) SINS Score: IG 6.6: CG 7.3 **Bone modifying agent:** IG 8 (66%) CG 4 (50%) **SREs:** Surgery: NR SCC: NR RT: NR Fractures: NR	**SAE exercise related:** 0 **SAE non-exercise related:** 0 **Non-SAE exercise related:** 0 **Non-SAE non-exercise related:** 0
Dreyling et al. (2025) [25]	Germany	RCT	December 2020 – November 2021	32 participants (16 IG: 16 CG) Newly diagnosed receiving VCd **Time diagnosis:** NR **Male**: IG *n* = 11 (69%): CG *n* = 11 (69%) **Age:** Mean: IG 67.3yrs: CG 61.1yrs	**MMBD:** IG *n* = 13 (81.3%): CG *n* = 14 (87.4%) **Bone modifying agent:** NR **SREs:** Surgery: NR RT: NR SCC: NR Fractures: NR	**SAE exercise related:** 0 **SAE non-exercise related:** 5 x? **Non-SAE exercise related:** 0 **Non-SAE non-exercise related:** 0

Key: *RCT* Randomised controlled trial, *IG* Intervention group, *CG* Control Group, *MM* Multiple myeloma, *MMBD* Multiple Myeloma Bone Disease, *SINS* Spinal Instability Neoplastic Score, *Mths* months, *Yrs* years, *Wks* weeks, *SREs* Skeletal related events, *SCC* Spinal Cord Compression, *SAE* Serious Adverse Event, *AE* Adverse Event, *LBP* Lower Back Pain, *NR* not reported, *RT* Radiotherapy, Days: d, *VCF* Vertebral Compression Fracture VCd: bortezomib– cyclophosphamide–dexamethasone

**Table 2:** Characteristics of the intervention

Reference	Timing enrolment	Type	Intensity	Frequency/Setting	Duration	Clinician
Groenveldt et al. (2013)	Post MM treatment Time diagnosis: NR	Aerobic:(treadmill walking, stationary cycling) Resistance training: UL/LL (machine,bodyweight,bands) Stretching	Aerobic: 15 min bouts at intensity 50% of HRR Every 4 wks time 5 min & intensity 5% HRR Heart rate monitors Resistance: 1) Initiate lightest weight 3 × 10 reps 2) reps 3 × 15 3) weight 3 × 10 reps 4) reps 3 × 15 Home: BORG set class	Mths 1–3 (1 × supervised 2 × HEP per wk) Mths 4–6 (3 × wk HEP, 1 × mth supervised) HEP or supervised at centre	6 mths	Physio Experience: NR
Shallwani et al. (2015) [29]	During MM treatment Time diagnosis: NR	Aerobic: Low impact Resistance training	Aerobic: Low impact, low to mod intensity Resistance: low to mod intensity No details how progressed	Aerobic: 15–60 min daily Resistance: 2–3 × wk HEP or supervised at centre	NR	Initial ax: Physio F/Up: Trained Exercise clinician Experience: NR
Larsen et al. (2019) [21]	Newly diagnosed MM (1 week) Time diagnosis: NR	Aerobic: (stationary bike) Resistance training: 3 × exs upper body, 5 × exs lower body, 1 × ex truncus Stretching: 3 muscle groups PA: Patient preference	Aerobic: 20 min RPE 12–13 14–16 Resistance: 1) 30–45 min 3 × 12–15 reps 2) weight 3 × 10–12 reps Stretching: 5 min, 30 s static Indep PA: 10 min continuous to total 30 min RPE 12–13 14–16	3 × wk ex programme, supervised or unsupervised Indep PA—4 × wk	10 wks	Physiotherapist Experience: NR
Koutoukidis & Land et al. (2020) [9]	Stable MM disease for 6 wks Time diagnosis: NR	Aerobic: (treadmill walking, stationary cycling, cross trainer, stepper) Resistance training: UL/LL/Trunk (machine, bodyweight & resistance bands)	Aerobic: 50–75% maximum heart rate up to 30 min Every 4 wks time by 5 min & intensity by 5% maximum heart rate Resistance: 10-RM assessment	Mths 1–3 (1 × supervised 2 × HEP per wk) Mths 4–6 (3 × HEP per wk, 1 × supervised per mth) HEP or supervised at centre	6 mths	Physiotherapist Experience: NR
Wefelnberg et al. (2022) [30]	Before & during treatment Time diagnosis: NR	Training- endurance & exercise machines for the major muscle groups Symptom & side effect specific exercise modules	NR	NR	37wks (SD = ± 43.5) 58.1 sessions (SD = ± 75.2)	HCP: NR Experience: NR
Purdy et al. (2022) [27]	(i) Transplant ineligible, first-line treatment (ii) transplant eligible, > 3 months post-transplantation (iii) relapsed/recurrent myeloma with ≥ 1 prior line of treatment Time diagnosis: Median 35mths (range 9–164)	Aerobic: (walking, elliptical or cycling, dance) Circuit based—10 min aerobic warm up, 2 rounds 8 resistance exercise circuit (UL/LL/Core/Balance) 5 min stretch	Aerobic: wk 1–3 RPE 3, wks 4–9 RPE 3/4, wks 10–12 RPE 4 Started on 1 out of 4 programmes dependent on fitness Resistance: wks 1–3 RPE 3, wks 4–9 RPE 4, wks 10–12 RPE 5	wks 1–6 (virtually supervised 1 × wk 1 × HEP) wks 7–12 (virtually supervised 1 × wk 2 × HEP)	12 wks	Kinesiologist > 3 years of exercise oncology exp oversight—exercise physiologist & physio PI: Physiotherapist with 20 years in cancer rehabilitation
McCourt et al. (2023) [23]	MM transplant eligible Time diagnosis: Median (IQR) IG 7mths (6,11) CG 6mths (5,10)	Aerobic: (Treadmill walking or stationary cycling in gym setting; walking, or use of own exercise machine) Resistance: mutli joint functional exs (UL/LL/Core)	Aerobic: Start 15 min, every wk time by 5 min. Intensity 60–80% HRR Resistance: Intensity determined 10-rep max	Pre admission: 1 × supervised 2 × HEP Admission: remote telephone support Rehab: 3 × wk HEP, 1 × wk telephone support HEP or supervised at centre COVID changed to virtual classes	Pre admission: 6-8wks Admission: 2-3wks Post discharge: 12 wks	Physiotherapist Experience: MSK, oncology, group class
Nicol et al. (2023)	Any stage MM (i) First line, ASCT eligible, (ii) First line, ASCT noneligible (iii) Relapsed (iv) In remission Time diagnosis: NR	Aerobic—cycling, walking Resistance—mutli joint functional exercises (UL/LL/Core) Impact (i.e., marching, stomping, jumping, or drop jumping from a height)	Aerobic: Intensity 60–80% peak HR (RPE 12–15), 2 × 4-min bouts at > 85% peak HR (RPE 16–19) 20 min Resistance: 6–10 reps 2–3 sets. Intensity determined by 6–10 rep max. Target intensity 60–80% Impact: ≥ 80 reps, ≥ 160 impacts per session	Mths 0–3 (2 × supervised 1 × HEP per wk) Mths 3–6 (3 × HEP per wk, offer of 1 × supervised per wk) Mths 6–12 (self directed HEP) HEP or supervised at centre or remote	12 mths	Exercise physiologist Experience: MSK, oncology, group class
Dapunt et al. (2023) [28]	Any stage MM Time diagnosis: Median 25.5mths (range 2–159)	Body Awareness Resistance (focused strength core & LL) Coordination Stretching & PA encouraged	Aerobic: moderate intensity Resistance: 3–4 mths progressed core strengthening eg rotation spine	Aerobic: 150 min per wk Resistance: 2–3 × per wk HEP supported by exercise therapy mobile app or 1:1 supervision local therapist	Progressed at 3–4 mths Follow up 9 mths	Exercise therapist under orthopaedic supervision or 1:1 supervision by a local therapist Experience: NR
Larsen et al. (2024) [22]	Newly diagnosed MM (1 week) Time diagnosis: NR	Aerobic: (stationary bike) Resistance training: 3 × exs upper body, 5 × exs lower body, 1 × ex truncus Stretching: 3 muscle groups PA: Patient preference	Aerobic: 20 min RPE 12–13 14–16 Resistance: 1) 30–45 min 3 × 12–15 reps 2) weight 3 × 10–12 reps Stretching: 5 min, 30 s static Indep PA: 10 min continuous to total 30 min RPE 12–13 14–16	3 × wk ex programme, supervised or unsupervised Indep PA—4 × wk	10 wks	Physiotherapist Experience: NR
Kollikowski et al. (2025) [20]	Any stage MM Time diagnosis: NR	Impact: marching, steps ups, hopping, jumping, high jumping, long jumping, jumping down height, one-legged jumping	Every 4 wks—new exs or intensity (number of jumps, height or distance, & complexity of the exercise)	Impact: 2 × wk supervision 1 × wk HEP HEP or supervised at centre or remote	6 mths	HCP: NR Experience: NR
Dreyling et al. (2025) [25]	Newly diagnosed receiving VCd Time diagnosis: NR	Aerobic exercise Resistance training	Aerobic: HR over 100 BPM Resistance: NR	2 × wk resistance training supervised on therapy days D1, D8, D15 each cycle then unsupervised remaining days 150 min aerobic training over 1 week	3 mths	HCP: NR Experience: NR

Key: *MM* Multiple Myeloma, *NR* not reported, *UL* Upper Limb, *LL* Lower Limb, *HRR* Heart Rate Reserve, *Wk* Week, *Mths* months, *HEP* Home exercise programme, *F/up* Follow up, *Ax* Assessment, *PA* Physical Activity, *RPE* Rate of perceived exertion, *MSK* Musculoskeletal, *HCP* Health care professional, *PI* Principle investigator, *Ex* exercises, *Indep* Independent, *IG* intervention group, *CG* Control Group, *ASCT* Autologous Stem Cell Transplant, *mins* minutes, *sec* seconds

**Table 3:** Assessment procedures and exercise adaptions utilised

Reference	Assessment	Exclusion criteria	Frequency exercise adaption	Reason for exercise adaption	How adapted exs
Groenveldt et al. (2013)	MM clinic screen: stable disease, skeletal survey, bloods, ± ECG Imaging—assess fracture risk in MDT Large lytic lesions in long bones, extensive lytic lesions referred surgery ± RT	Bone related: Spinal instability, risk of fracture, MSK limited mobility Other: Erythropoietin, unstable angina	NR	Modified as needed eg spinal deformity or LBP Not modified for stable fractures	Tailored to individual Changed: standing to sitting
Shallwani et al. (2015) [29]	Medical history (disease status, painful bone lesions, history SREs, renal dysfunction, amyloidosis) Physiotherapist—demographics, medical history, imaging	Nil	NR	Activity levels & symptoms	Tailored to individual
Larsen et al. (2019) [21]	Haematologist: Imaging: site, size, pain ± time from fracture, assessed by MIRELs	Bone related: Spinal cord compression, spinal instability (SINS score > 12) Other: Untreated cardiac failure or arrhythmia, severe chronic cardiac failure (NYHA 3–4), other severe comorbidities that would not permit physical exercise, psychological or psychiatric disorders	n = 12	Restrictions: Femur/Humerus: Osteolysis > 2/3 compacta or 1/3–2/3 with pain; moderate/functional pain with bone destruction Pelvis: Fracture or osteolysis > 2 cm in acetabulum or > 2/3 of rami. Ribs/Vertebrae: Fractures < 6 wks old. Former Fracture Site: Any pain	Tailored to individual Progression (*n* = 4); regression (*n* = 1), both progression & regression (*n* = 7) Principles by Galvão et al. (2011)—No weights or movement at involved site
Koutoukidis & Land et al. (2020) [9]	Doctor confirmation stable disease: imaging, ± ECG Imaging—assess fracture risk in MDT using MIRELs	Bone related: Spinal instability, recent surgery (< 4 wks) pathological fractures or spine, fracture risk (Mirel’s), MSK limited mobility Other: Abnormal ECG, enrolled exercise study, unstable angina, cognitive impairment	NR	NR	Tailored to individual
Wefelnberg et al. (2022) [30]	NR Bone assessment NR	Nil	NR	Ability	Tailored to individual
Purdy et al. (2022) [27]	Approved physician—based on stability of symptoms, pain optimisation	Bone related: Physician approved safe to exercise at home & bone disease taken into consideration Other: Diagnosis of amyloid light-chain amyloidosis, solitary plasmacytoma, Waldenstrom macroglobulinemia, too frail for HEP	*n* = 125	History of fracture/lytic lesion in area *n* = 11, disease-related pain *n* = 27 reduced range of movement *n* = 19 high strength *n* = 13, non myeloma pain *n* = 10, low strength *n* = 9, high endurance *n* = 8, equipment *n* = 8, more appropriate *n* = 8, neuropathy *n* = 7, fatigue *n* = 5	Tailored to individual Exercise alternative *n* = 33, reduced intensity *n* = 28, custom routine *n* = 24, increase intensity *n* = 15, exercise order *n* = 9, exercise addition *n* = 6, exercise removal *n* = 9 Principles—International Bone Metastases Exercise Working Group- Avoidance of exs with high fall risk, extreme spinal movements, rapid and/or weight-loaded end-range Emphasis-technique, alignment, tempo
McCourt et al. (2023) [23]	Screened inclusion/exclusion—discussion with MDT Bone assessment NR	Bone related: Spinal instability, SCC, neurological deficits, recent surgery (< 6 wks) for pathological fractures or spine Other: Abnormal ECG, unstable angina, enrolled exercise study, unable to consent, Declined	NR	Ability, presence MMBD, symptoms	Tailored to individual
Nicol et al. (2023)	Location of lesions provided by treating clinician & monitored by exercise physiologist	Bone related: No MSK complaints Other: No neurological, respiratory, metabolic, cardiovascular conditions preventing safe exercise completion. Unable to consent/attend	88%	Co-morbidities, injuries, illnesses, relative/absolute contraindications, ability, MMBD	Tailored to individual Pelvic/Axial/Lower limb lesions no impact—isometric Reduced intensity/duration; removal/replacement of specific exercises Rief et al. (2014) Hart et al. (2018)
Dapunt et al. (2023) [28]	Orthopaedic specialist reviewed imaging (whole body CT in last ≤ 3mths) using SINS and MIRELs Individual personal performance	Nil	NR	Early stages of the disease and/or with new SRE; persistently high disease activity (≥ partial response); no signs osteosclerosis	Tailored to individual Reduced programme
Larsen et al. (2024) [22]	Haematologist—Imaging: site,size, pain ± time from fracture, assessed by MIRELs	Bone related: Spinal cord compression, spinal instability (SINS score > 12) Other: Untreated cardiac failure or arrhythmia, severe chronic cardiac failure (NYHA 3–4), other severe comorbidities that would not permit physical exercise, psychological or psychiatric disorders	NR	Restrictions: Femur/Humerus: Osteolysis > 2/3 compacta or 1/3–2/3 with pain; moderate/functional pain with bone destruction Pelvis: Fracture or osteolysis > 2 cm in acetabulum or > 2/3 of rami. Ribs/Vertebrae: Fractures < 6 wks old. Former Fracture Site: Any pain	Tailored to individual Progression (*n* = 4); regression (*n* = 1); both progression & regression (*n* = 7) Principles by Galvão et al. (2011)—No weights or movement at involved site
Kollikowski et al. (2025) [20]	(SINS) before initiating the intervention	Bone related: Fractures ≤ 12 mths or spinal instability (SINs ≥ 11excluded impact exercise) Other: Nil specified	*n* = 175	Pain or discomfort	Tailored to individual Progression (*n* = 143 46.1%); Regression (*n* = 32 10.3%)
Dreyling et al. (2025) [25]	Assessed by physicians of different disciplines by interdisciplinary myeloma tumor board Freiburg	Extensive, unstable bone lesions, with a tendency to fracture or inability to walk	NR	NR	patient general condition and MM-specific conditions

Key: *MM* Multiple myeloma, *MDT* Multidisciplinary Team, *MMBD* Multiple Myeloma Bone Disease, *SINS* Spinal Instability Neoplastic Score, *Mths* months, *Wks* Weeks, *SREs* Skeletal related events, *ECG* Electrocardiogram, *NR* Not reported, *RT* Radiotherapy, *SCC* Spinal Cord Compression, *MSK* Musculoskeletal, *LBP* Lower back pain, *HEP* Home Exercise Programme, *Exs* Exercises

**Table 4:** Outcomes of studies

Reference	Outcomes	Results of bone related outcomes
Groenveldt et al. (2013)	***Feasibility*** (accrual rate, screen pass rate, % completing programme), *Safety* (adverse events), *Acceptability* (adherence to programme, attendance of classes), *QoL* (FACT-G), *Fatigue* (FACIT-F), *Fitness* (8 min treadmill test), *Anxiety and depression* (HADS), *Body composition* (body mass, height, whole body fat and lean tissue), *Strength* (hand grip/knee extensor 10RM)	Nil
Shallwani et al. (2015) [29]	***Compliance*** (patient reported exercise details), *Fatigue* (VAS 0–10), *Physical Activity levels* (patient reported exercise details)	Nil
Larsen et al. (2019) [21]	***Feasibility and safety of intervention*** (attendance, adherence, tolerability, attrition, adverse events)**, *Feasibility and safety of physical tests*** (completion rates, adverse events), *Feasibility of participation* (eligibility, acceptance, attrition, decline, non-eligibility)	Nil
Koutoukidis & Land et al. (2020) [9]	***Fatigue*** (FACIT-F), *QoL* (FACT-G), *Fitness* (VO2 peak), *Anxiety and depression* (HADS), *Body composition* (weight, % body fat, muscle mass), *Strength* (hand grip/knee extensor 10RM), *Safety* (adverse events), *Physical Activity* (accelerometer)	Nil
Wefelnberg et al. (2022) [30]	*Exercise Adherence*, *Safety* (adverse events), *Attrition*, *Endurance capacity* (bike or cross-walker quasi ramp protocol), *Strength* (8RM at various strength exercise machines), *QoL* (EORTC QLQ C30), *Fatigue* (MFI), *Physical Activity* (GPAQ), *Anxiety and depression* (HADS)	Nil
Purdy et al. (2022) [27]	***Feasibility*** (recruitment rates, safety, adherence), *Satisfaction*, *Physical Fitness/Strength* (2 min step test, 30 s STS, Plank endurance test, One leg stand) *Upper body flexibility* (shoulder ROM), *Lower body flexibility* (modified sit and reach test), *QoL* (FACT MM), *Fatigue* (FACIT F), *Edmonton symptom burden*, *Neuropathy* (GOG-NTX4), *FACT bone pain*	Nil
McCourt et al. (2023) [23]	**Feasibility** (recruitment rate, ineligibility, attrition, adherence, adverse events), *Adherence* (attendance of programme), *Fatigue* (FACIT-F), *QoL* (FACT-G, EORTC-QLQ-C30), *Physical Activity* (IPAQ -SF), *Functional capacity* (6MWT), *Muscle strength* (TSTS, HGS), *Length of stay*, *Physical Activity & sedentary levels* (accelerometer), *Health Service Utilisation*	Nil
Nicol et al. (2023)	***Health related quality of life*,** *Safety* (adverse events), *Feasibility* (eligibility/uptake/), *Attrition, Attendance/adherence, Acceptability* (PACES-8)	Nil
Dapunt et al. (2023) [28]	Physical Activity behaviour, Pain	Nil
Larsen et al. (2024) [22]	***Knee extension strength*** (dynamometer), *Physical Function* (6MWT) Strength (30 s TSTS, HGS), *QoL* (EORTC-QLQ-C30), *Pain* (BFI), *Physical Activity* (Patient diary, daily steps—accelerometer), *BMD* (DEXA scans)	BMD lumbar (g/cm2) (IG T1 1.00 T3 1.02 (*p* = < 0.001) T4 1.02 (*p* = < 0.001) (CG T1 0.88 T3 0.94 (*p* = < 0.001) T4 0.99 (*p* = < 0.001) BMD whole body (g/cm2) (IG T1 0.94, T3 0.98 (*p* = 0.128) T4 0.97 (*p* = 0.056) (CG T1 0.96 T3 0.97 (*p* = 0.053) T4 0.99 (*p* = 0.001) BMD Hip (g/cm2) (IG T1 0.85 T3 0.88 (*p* = 0.456) T4 0.88 (*p* = 0.707) (CG T1 0.83 T3 0.84 (*p* = 0.598) T4 0.84 (*p* = 0.345)
Kollikowski et al. (2025) [20]	**Feasibility (adherence/drop outs), Safety (adverse and serious adverse events),** *QoL* (EORTC QLQ C30) and MY20), *Physical Function* (6MWT and Chair rise)	Decrease Values: AP (IG, 80 to 17.5 U/l, *P* = 0.004; CG, 71 to 18.5 U/l, *P* = 0.016) P1NP (IG, 37.5 to 24.3 µg/l, *p* = 0.82; CG 41 to 37.6 µg/l *p* = 0.031) sRANKL (IG 528.48 to 244.6 pmol/l, *P* = 0.547; CG 96 to 67 pmol/l, *P* = 0.938) RANKL/OPG ratio (IG 142.91 to 36.22 p = 0.164; CG 24.93 to 15.53 *P* = 0.813) Increase Values: PTH (IG 41 to 65.6 ng/l, *P* = 0.039; CG, 24 to 41.9 ng/l, *P* = 0.031) OPG (IG 4.04 to 4.58 pmol/l *p* = 0.203: CG 4.54 to 5.16 pmol/l. *p* = 0.219) CTX (IG 0.03 to 0.04 ng/ml *p* = 1.00; CG 0.1 to 0.11 ng/ml *p* = 0.313) Sclerostin (IG 31.34 to 35.44 pmol/l *p* = 0.91; CG 40.7 to 39.98 pmol/l *p* = 0.156) BAP (IG 9.4 to 10.3 µg/l p = 0.496; CG 7.8 to 8.2 µg/l *p* = 0.656) 56 lesions ≥ 5 mm diameter (IG:30 impact, CG stretching:26) Size of lesions—Sum lesion score unchanged both groups
Dreyling et al. (2025) [25]	**Safety (Serious adverse events, bone fractures, SREs) Feasibility (participant willingness to participate, compliance of programme),** EFS (time from randomization to progressive disease, dose reduction, haematological adverse effects) R-MCI, functional tests (Grip strength, TUGT), QoL (SF12), Depression (HDRS17), biomarkers (Albumin, HDL, LDL, CRP, CK, proBNP), weight, Body Mass Index, comorbidities (osteolytic lesions, fatigue, depression recurrent infections, anaemia), AEs (treatment tolerance, hospitalisation, response to therapy)	Nil

Key: *QoL* Quality of Life, *FACT-G* Functional Assessment of Cancer Therapy- General, *FACIT-F* Functional Assessment of Chronic Illness Therapy – Fatigue, *HADS* Hospital Anxiety and Depression Scale, *MFI* Mutlidimensional Fatigue Inventory, *GPAQ* Global Physical Activity Questionnaire, *VAS* Visual Analogue Scale, *EORTC-QLQ-C30* European Organization for Research and Treatment of Cancer Quality of Life Questionnaire, *ROM* Range of Movement, *FACT MM* Functional Assessment of Cancer Therapy – Multiple Myeloma, *GOG-NTX4* Functional Assessment of Cancer Therapy—Gynecologic Oncology Group-Neurotoxicity 4, *HGS* Hand grip, *IPAQ -SF* International Physical Activity Questionnaire – Short Form, *6MWT* Six Minute Walk Test, *TSTS* Timed Sit to Stand, *BFI* Brief Pain Inventory, *BMD* Bone Mineral Density, *MY20* European Organization for Research and Treatment of Cancer Quality of Life Questionnaire Myeloma Module, *IG* Intervention Group, *CG* Control Group, *AP* alkaline phosphatase, *P1NP* N-terminal propeptide of human procollagen type I, *sRANKL* serum receptor activator of nuclear factor kappa-Β ligand, *OPG* osteoprotegerin, *CTX* C-terminal telopeptide of type I collagen, *BAP* bone specific alkaline phosphatase, *PTH* parathyroid hormone, *SREs* Skeletal related events, *HDL* high-density lipoprotein, *LDL* low-density lipoprotein, *CRP* C-reactive Protein, *CK* creatine kinase, *proBNP* brain natriuretic peptide, *R-MCI* Revised Myeloma Comorbidity Index, *TUGT* timed-up-and-go test, *HDRS17* Hamilton Depression Rating Scale containing 17 items, *AEs* adverse events, *EFS* event free survival

Data was presented in tabular form and discussed narratively through text.

## Results

**Fig. 1:**
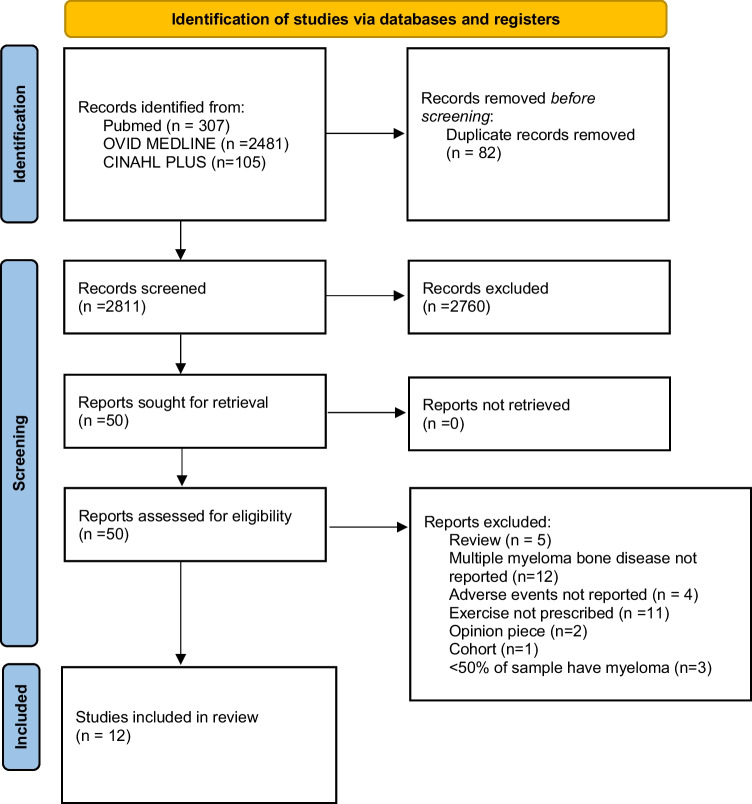
PRISMA flow chart

The search strategy yielded 307 records from PUBMED, 2481 from OVID-MEDLINE and 105 records from CINAHL PLUS producing a total of 2893 records. After importing all records into EndNote, 82 duplicates were removed, leaving 2,811 titles and abstracts for screening by JL. Following application of the inclusion and exclusion criteria, 50 articles were retained for full-text review. Subsequently, 38 publications were excluded, yielding 12 publications for inclusion in this scoping review. A detailed description of the screening process is presented in the PRISMA flow chart (Fig. 1) [18].

## Characteristics of the included studies

An RCT design was employed in 7 studies [9, 20–25], while 2 studies adopted a single arm pre–post design [26, 27]. A retrospective cohort design was used in 3 studies [28–30]. Study locations included Germany (4 studies) [20, 25, 28, 30], United Kingdom (3 studies) [9, 23, 26], Denmark (2 studies) [21, 22], Canada (2 studies) [27, 29] and Australia (1 study) [24]. All studies included MMBD participants, though none exclusively targeted this subgroup.

Across 654 myeloma participants, 475 were allocated to exercise interventions, of whom 346 had MMBD. The reporting of MMBD varied substantially across studies. Just over half of the studies reported MMBD as the percentage of participants with bone involvement, with prevalence typically ranging from approximately 69–86% [9, 20–23, 25, 27]. Other studies defined MMBD based on the presence of lesions [24, 28, 29], focal or disseminated lesions [30], significant MMBD (which they defined as vertebral compression fractures or fractures in long bones) [26], or osteoporosis/osteopenia [24]. Several studies provided additional detail regarding lesion burden [23, 29], anatomical distribution broadly (e.g. axial or peripheral) [23] or specifically (e.g. cervical, thoracic, lumbar spine; ribs; pelvis; femur; humerus; skull; and other areas) [24, 27], or stability scores (e.g. Mirels or Spinal Instability Neoplastic Score (SINS)) [20, 28]. Although most studies reported a history of SREs, comprehensive reporting of all SREs was uncommon.

Bone-modifying agents were reported in 3 RCTs. Bisphosphonates were utilised in 2 studies (zoledronic acid *n* = 7, 77% [20]; overall prevalence 82.8% [24]. RANKL inhibitor Denosumab use was also reported (*n* = 39, 89% [22]; *n* = 1, 11% [20]. Characteristics of the included studies are found in Table 1.

## Safety—adverse events

Across the 12 included studies, 346 MMBD participants engaged in exercise programmes. Exercise-related AEs didn’t occur very frequently and 7 AEs were reported across 4 studies [9, 22, 23, 27]. These included 1 case of hip pain during a home exercise programme [9], 4 cases of back pain (mild back pain following low-impact jumping jacks, back pain related to seated bridging, mild to moderate back pain after bridging, and moderate to severe back pain following a hip flexor stretch) [27], 1 episode of dizziness during a supervised exercise class [23] and 1 AE of unspecified nature attributed to excessive load [22].

Two studies reported outcomes related to these exercise-related AEs. In the study reporting hip pain, symptoms resolved spontaneously [9], while in the study reporting 4 cases of back pain, exercise was paused for a few days up to 7 days [27]. However, none of the studies explicitly reported whether these AEs occurred in MMBD participants.

Non–exercise-related AEs were reported more frequently, with 14 events across 5 studies [9, 21, 22, 24, 27]. These included pain (*n* = 1), dizziness (*n* = 1; cause not reported) [21], back pain (*n* = 4; cause not reported) [9], spinal fracture following a fall at home (*n* = 1) [27], lower back pain due to a slip on ice (*n* = 1) [27], arrhythmia in a participant with a history of cardiac intervention (*n* = 1) [27], and a hypotensive episode while standing still during an exercise class (*n* = 1) [24]. One study reported four additional AEs without specifying their nature [22]. The majority of studies reported outcomes following AEs, which ranged from continuation of the exercise programme [24], to temporary pauses in the intervention [21, 22, 27], and discontinuation in cases of more severe AEs (e.g. spinal fracture) [27].

Overall, these findings suggest exercise-related AEs were infrequent, mostly mild, and generally resolved without long-term effects. However, no studies reported whether AEs occurred specifically in MMBD participants, and the causes and outcomes of AEs were not consistently reported. AE reporting across studies is summarised in Table 1.

## Exercise considerations

### Assessment to initiate exercise

Seven studies reported that exercise participation was screened or approved by a haematologist [21, 22], Doctor [9, 25–27], or in a real-world clinical setting orthopaedic specialist [28]. Just over half of the studies (*n* = 7) explicitly assessed fracture or instability risk prior to enrolment [9, 20–22, 25, 26, 28], with 5 studies using validated tools such as Mirels or SINS [9, 20–22, 28].

Three studies described systematic bone assessments led using imaging modalities including (CT/X-ray) [21, 22, 28]. In 2 studies by Larsen et al. [21, 22], lesion characteristics, pain, time since fracture, and Mirels scores were used to guide exercise restrictions [21, 22], while Dapunt et al. (2023) individualised exercise prescription based on recent whole-body CT imaging, SINS/Mirel scores, performance measures, and were the only study to consider haematological treatment response [28].

Additional screening approaches included physician approval based on symptom stability and pain control [27], and multidisciplinary medical screening involving imaging, ECG, and disease stability assessments [9, 25, 26]. In 2 studies, skeletal surveys were reviewed by multidisciplinary teams, with high-risk cases (large lytic lesions or extensive pelvic disease) referred for surgical or radiotherapy assessment [9, 26]. Other studies reported variable or limited screening procedures, including medical teams providing lesion/complication details to exercise physiologists who then decided on appropriate exercise management [24], physiotherapist review of imaging and medical history [29], or acknowledgement of bone disease without further assessment detail [23].

### Eligibility

The exclusion criteria for the included studies have been reported according to the type of exercise prescribed and are summarised in Table 3. Real-world evaluations (3 × retrospective) did not state any exclusion criteria [28–30].

#### Aerobic/strength/stretching exercises

Six studies who prescribed aerobic/strengthening and stretching exercises excluded participants with spinal instability [9, 20–23, 26], with Larsen et al. (2019, 2024) specifying a SINS score > 12 [21, 22]. Three studies excluded participants with spinal cord compression [21–23], Three studies excluded individuals at risk of fracture, [9, 25, 26] determined by 1 study using the Mirels score, though no cut-off was provided [9]. Additionally, 2 studies excluded patients who were less than 4 or 6 weeks post spinal or other fracture-related surgery [9, 23]. Three studies excluded patients with any musculoskeletal condition [9, 24, 26]; in 2 of these, exclusion was based on reduced mobility [9, 26]. Purdy et al. (2022) reported a physician ensured the patient was safe to exercise and assessed bone disease burden but didn’t report what this entailed [27].

#### Impact exercises

Two studies provided impact exercises [20, 24]. One study excluded patients who had spinal instability (SINS ≥ 11) or were less than 12 months post-fracture [20]. The other impact study excluded patients with musculoskeletal conditions and placed restrictions on impact exercise if patients had pelvic, lumbar or lower limb lesions present [24].

Overall, for aerobic, resistance, and stretching programmes, participants were commonly excluded if they had spinal instability, spinal cord compression, a recent fracture or surgery, or were deemed at risk of fracture. Studies prescribing impact exercise applied more stringent criteria, with exclusions based on structural bone integrity (e.g. no lesions in load-bearing regions) and longer time since fracture.

### Timing to initiate exercise and type of exercise delivered

Exercise interventions were initiated at varying points along the myeloma treatment pathway, ranging from diagnosis through to post completion of treatment. Table 2 summarises intervention characteristics.

Early-pathway interventions (5 studies), delivered alongside initial chemotherapy, focused mainly on aerobic and strength exercises, with 1 programme incorporating fatigue and gait training [21, 22, 25, 29, 30]. All early interventions used a mix of supervised and home-based sessions [21, 22, 25, 29, 30].

Prehabilitation programmes (1 study) typically began post-induction and continued into the post-transplant period, combining supervised and home-based aerobic and resistance training [23].

Remission phase programmes (2 studies) were delivered over longer durations with gradually reduced supervision, supporting ongoing independent aerobic, resistance, and stretching exercise [9, 26].

Where interventions spanned all treatment stages (4 studies), most participants had undergone some form of treatment [20, 24, 27, 28]. Two studies reported time from diagnosis (median 25.5 months; range 2–159) [28] and (median 35 months; range 9–164) [27]. In 1 study, most exercise participants were in maintenance (*n* = 5) or treatment-free phases (*n* = 4), with a smaller group post-transplant (*n* = 2) [20]. In the other, the exercise cohort largely comprised transplant-eligible (*n* = 8), relapsed (*n* = 8), or remission patients (*n* = 8) [24]. Interventions across these studies commonly combined supervised and unsupervised aerobic, strength, and stretching programmes, with 2 including impact-based exercise. Two studies were app-based [27, 28].

Overall, programmes span from diagnosis to maintenance/relapse stages in treatment and frequently incorporated aerobic, resistance training, with stretching or impact elements in some cases. Frequency ranged from 1–3 weekly sessions over 10 weeks to 12 months, with supervision often higher initially and tapering over time.

### Exercise adaptions

All the studies reported adapting exercise programmes to individuals needs [9, 20–30]. However, only 4 studies reported how many sessions were adapted: *n* = 12 [21], *n* = 125 [27], 88% [24] and *n* = 175 [20]. Table 3 provides details on the frequency, reasons, and methods of adaptation.

Reasons for adaptation varied but were most commonly related to MMBD, patient symptoms, or functional ability. Notably, Dapunt et al. (2023) was the only study to adapt programmes based on haematological parameters, including early-stage disease, new SREs on imaging, persistently high disease activity (≥ partial response), and absence of osteosclerosis [28].

Five studies described how exercises were adapted: through progression or regression [20–22, 24, 27] removal or alternative exercise [24, 27] or addition of exercise or change of order [27].

Five studies reported drawing on established exercise principles and guidelines for bone metastases, including 2 citing Galvão et al.(2011) [21, 22, 31], 1 using the International Bone Metastases Exercise Working Group (IBMEWG) guidelines (2022) [27, 32] and 1 study using Rief et al. (2014) [33] and Hart et al. (2018) [24, 34](Table 3). One study reported reducing the exercise programme, although the method was not specified [28].

Overall, all studies reported tailoring exercises to individual needs. However, infrequent reporting of adaptation frequency, rationale, methods, and consideration of haematological parameters limits translation into clinical practice.

### Health care professional

A range of health professionals were involved in prescribing and delivering exercise programmes across the included studies (see Table 2). Physiotherapists were the most commonly reported providers, delivering or prescribing exercise in 7 studies [9, 21–23, 26, 27, 29]. Exercise physiologists [24], exercise specialists [28, 29] and kinesiologists [27] were also reported, though the degree of their involvement varied between studies.

In several studies, prescribing and delivery were shared across professions or supported by multidisciplinary oversight [27–29]. However, reporting of professional qualifications and experience was limited, with 9 studies not providing this information [9, 20–22, 25, 26, 28–30].

### Outcomes specific to change in MMBD

None of the studies primary endpoint related to changes in MMBD and were therefore not powered to detect change, however, 2 studies reported on bone outcomes and details can be found in Table 4.

Kollikowski et al. (2025) investigated the effects of impact exercise on bone metabolic and structural parameters before the intervention and 6 months later [20]. They observed in the intervention group (IG) (12 patients) and control stretching group (SG) (8 patients) significant decreases in alkaline phosphatase (AP) (IG: 80 to 17.5 U/l, *P* = 0.004; SG: 71 to 18.5 U/l, *P* = 0.016) and in the stretching group significant decreases in N-terminal propeptide of human procollagen type I (P1NP) (IG, 37.5 to 24.3 µg/l, *p* = 0.82; CG 41 to 37.6 µg/l *p* = 0.031) [20]. Significant increases in parathyroid hormone (PTH) (IG: 41 to 65.6 ng/l, *P* = 0.039; SG: 24 to 41.9 ng/l, *P* = 0.031) but no significant changes in other bone marrow turnovers or blood parameters [20].

To assess changes in bone structure and mineralization, the same study detailed how low-dose CT scans were performed on all participants both before and after the 6 month intervention [20]. Up to 5 bone lesions measuring ≥ 5 mm in diameter were evaluated per participant. They calculated a sum lesion score based on the ratio of lesion sizes post- versus pre-intervention, with a ≥ 20% change considered clinically significant [20]. In total, 56 lesions were identified (IG:30 impact, SG stretching:26). However, no differences were observed in the sum lesion scores before and after the intervention for both groups [20].

Larsen et al. (2024) used DEXA scans to assess bone mineral density (BMD) [22]. They observed a significant increase in lumbar spine BMD within group differences in both the intervention (IG) (44 patients) and control groups (CG) (42 patients) at both 6 and 12 months (IG: T1 1.00 6mths 1.02 (*p* = < 0.001) 12mths 1.02 (*p* = < 0.001) (CG T1 0.88 6mths 0.94 (*p* = < 0.001) 12mths 0.99 (*p* = < 0.001) [22]. Additionally, whole-body BMD significantly increased in the control group at 12 months (CG T1 0.96 6mths 0.97 (*p* = 0.053) 12mths 0.99 (*p* = 0.001) but not the intervention group (IG T1 0.94, 6mths 0.98 (*p* = 0.128) 12mths 0.97 (*p* = 0.056) [22]. However, hip BMD remained unchanged in both groups over the 12 months (IG T1 0.85 6mths 0.88 (*p* = 0.456) 12mths 0.88 (*p* = 0.707) (CG T1 0.83 6mths 0.84 (*p* = 0.598) 12mths 0.84 (*p* = 0.345) [22].

## Discussion

This scoping review summarises the literature on exercise interventions in MMBD participants, mapping safety, assessment procedures, intervention characteristics, outcomes specific to change in MMBD, and research gaps. While all studies included participants with MMBD, none focused exclusively on this subgroup, and only 2 reported secondary outcomes related to exercise effects on bone health. Across studies, exercise appeared generally feasible and safe for MMBD participants under certain exclusion criteria, but reporting on MMBD characteristics, safety, assessments, and exercise adaptations was variable and inconsistent.

Previous reviews deemed exercise safe in myeloma patients but did not report specifically on MMBD [13]. This review finds that exercise-related AEs were infrequent, mostly mild, and generally resolved without long-term effects. However, none specified whether the 7 exercise-related AEs occurred in MMBD participants, and few reported outcomes. Luo et al. (2022) highlight that high-risk patients, such as those with MMBD, may raise safety concerns for clinicians and emphasize the need for clearer AE reporting in cancer exercise trials [35]. Future trials should report exercise and non-exercise AEs, specify causes, indicate which participants had MMBD, and describe outcomes. Thus, enabling clinicians to confidently assess the potential risks of exercise interventions for MMBD patients.

MMBD classification varied widely, from simple counts of affected participants to detailed descriptions of lesion type, location, extent, and SRE history, making comparisons and safety assessments difficult. Patients with a history of SREs show higher non-compliance to exercise [29] and poorer mobility and quality of life [6, 7], emphasizing the importance of detailed MMBD reporting when evaluating exercise interventions. Consistent reporting of SRE history, lesion type and location, bone-modifying agents use, and disease extent is recommended to support outcome evaluation and support clinicians in confidently recommending exercise for this potentially higher-risk group.

All studies reported exercise adaptations, but few detailed frequency, rationale, or specific modifications for MMBD, and only a small number used established bone metastases guidelines [31–34], limiting clinical translation. Most studies prescribed aerobic and strengthening exercises, supporting safety in MMBD, while 2 included impact exercises with stricter bone disease criteria [20, 24]. Supervised sessions were common, often combined with home-based training, aligning with IBMEWG recommendations to start supervised and transition to independent exercise [32]. Physiotherapists most commonly prescribed exercise, followed by exercise physiologists and kinesiologists, though few reported practitioner qualifications as recommended by IBMEWG [32]. Improved reporting of qualifications and exercise adaptation methods including frequency and rationale are needed to strengthen clinical guidance.

No trials investigated bone changes as a primary outcome. Two assessed bone as a secondary outcome using bone turnover markers and imaging, with no significant effects [20, 22], likely due to brief interventions (≤ 10 weeks) [20]. Evidence from peri- and postmenopausal women suggests interventions longer than 6 months can improve femoral neck BMD [36]. Additionally, a sufficiently high level of mechanical stress is required to stimulate bone adaptation [36]. These findings highlight a research gap, as the optimal type, intensity, and duration of exercise needed to elicit bone mass changes in MMBD patients remains unclear.

Participants were recruited at all disease stages, with most studies performing medical screening as recommended for bone metastases patients by the IBMEWG [32]. Studies commonly excluded patients with spinal instability; fewer excluded those with spinal cord compression, limited mobility, fracture risk, or recent surgery. Imaging and tools such as SINS or Mirels scores were frequently used, but assessment details and consistency varied. Notably, only 1 study considered haematological parameters [28]. Evidence suggests that sclerosis of bone lesions on imaging can occur across different disease responses and treatment stages, highlighting the need to record these parameters in future studies [37]. Improved reporting of haematological and imaging data and clearer documentation of screening processes would help guide clinical decision-making and appropriate timing of exercise, which in current studies was largely based on treatment stage rather than clinical factors.

## Strengths and limitations

To our knowledge, this is the first review to map the safety, outcomes, assessment, and intervention characteristics of exercise studies in myeloma patients with a specific focus on bone disease. Strengths include a comprehensive search strategy and use of an established review framework. As a scoping review, we did not assess methodological quality or risk of bias, limiting conclusions regarding evidence robustness. Additionally, exclusion of grey literature may have resulted in omission of relevant data.

## Future directions and recommendations

The lack of exercise studies specifically targeting MMBD patients and limited evidence on exercise parameters for bone-specific or patient-reported outcomes highlight key research gaps. Future trials should report detailed MMBD characteristics, assessment procedures (including haematological and imaging parameters), exercise-related AEs in MMBD patients, and exercise adaptations. Clear reporting of adaptation frequency and rationale is essential for clinical applicability and generalisability. In the absence of MMBD-specific exercise guidance, exercise prescription currently draws on bone metastases guidelines; development of consensus-based MMBD exercise guidelines may improve assessment, prescribing confidence, and safe exercise delivery for this higher-risk population.

## Conclusion

This scoping review identified current evidence and key gaps in exercise studies including patients with MMBD. Reported exercise-related AEs were infrequent and generally mild without long term consequences, supporting safety, although most studies did not specify whether events occurred in MMBD participants. Medical clearance was usually sought, with limited consideration of haematological parameters. Aerobic and resistance programmes were commonly prescribed and adapted, but inconsistent reporting of MMBD characteristics, assessment methods and exercise modifications limits clinical translation. Future trials should clearly document MMBD features, screening procedures, exercise adaptations and AEs. Dedicated MMBD-focused studies are needed to determine optimal exercise prescriptions and effects on bone and patient-reported outcomes.

## Data Availability

Data is available upon request.

## Appendix

Search Strategy

OVID MEDLINE

7th October 2025

**Table:**

#	Searches	Results
1	exp Multiple Myeloma/	51549
2	exp Plyometric Exercise/or exp Exercise/or exp Exercise Therapy/or exp Circuit-Based Exercise/or exp Warm-Up Exercise/or exp Exercise Movement Techniques/or exp Cool-Down Exercise/	328217
3	exp Muscle Weakness/	10293
4	exp Musculoskeletal Physiological Phenomena/	1234437
5	exp Physical Examination/	1453605
6	(physiotherap$ or physical therap$).mp	100656
7	exp Muscle, Skeletal/	326488
8	exp Mind–Body Therapies/	51014
9	(Rehabilitation or Exercise or physical exercise or physical activity or Muscle weakness or Muscle fatigue or Musculoskeletal Physiological Phenomena or Physical Examination or Muscle strength or posture or physical fitness or aerobic exercise or run$ or jog$ or walk$ or weight train$ or resistance train$ or strength train% or physical activ$ or tai chi or yoga or balance or aerobic or work$ or physiother$ or physical therap$ or work$).mp. (mp = title, book title, abstract, original title, name of substance word, subject heading word, floating sub-heading word, keyword heading word, organism supplementary concept word, protocol supplementary concept word, rare disease supplementary concept word, unique identifier, synonyms, population supplementary concept word, anatomy supplementary concept word)	4260622
10	1 and 9	2481
